# Car Bumper Effects in ADAS Sensors at Automotive Radar Frequencies

**DOI:** 10.3390/s23198113

**Published:** 2023-09-27

**Authors:** Isabel Expósito, Ingo Chin, Manuel García Sánchez, Iñigo Cuiñas, Jo Verhaevert

**Affiliations:** 1 atlanTTic Research Center, Signal Theory and Communications Department, Universidade de Vigo, 36310 Vigo, Spain; iexpositop@uvigo.gal (I.E.); manuel.garciasanchez@uvigo.gal (M.G.S.); 2 IDLab, Department of Information Technology, Ghent University-imec, 9052 Ghent, Belgium; ingo.chin@ugent.be (I.C.); jo.verhaevert@ugent.be (J.V.)

**Keywords:** ADAS technologies, attenuation measurements, automotive radar, ITS technologies, material characterization, radio propagation, wireless

## Abstract

Radars in the W-band are being integrated into car bumpers for functionalities such as adaptive cruise control, collision avoidance, or lane-keeping. These Advanced Driving Assistance Systems (ADAS) enhance traffic security in coordination with Intelligent Transport Systems (ITS). This paper analyzes the attenuation effect that car bumpers cause on the signals passing through them. Using the free-space transmission technique inside an anechoic chamber, we measured the attenuation caused by car bumper samples with different material compositions. The results show level drops lower than 1.25 dB in all the samples analyzed. The signal attenuation triggered by the bumpers decreases with the frequency, with differences ranging from 0.55 dB to 0.86 dB when comparing the end frequencies within the radar band. Among the analyzed bumper samples, those with a thicker varnish layer or with talc in the composition seem to attenuate more. We also provide an estimation of the measurement uncertainty for the validation of the obtained results. Uncertainty analysis yields values below 0.21 dB with a 95% coverage interval in the measured frequency band. When comparing the measured value with its uncertainty, i.e., the relative uncertainty, the lower the frequency in the measured band, the more accurate the measurements seem to be.

## 1. Introduction

The automotive industry is evolving into increasingly autonomous-ready cars [1]. The agents of change are active safety systems based on cameras, radars, LIDARs (Laser Imaging Detection and Ranging) and ultrasonic sensors. They can detect people and objects in the surroundings of the vehicle, allowing its central processing system to react accordingly [2]. The advantage of radar sensors, which makes them more important compared to other systems, is their capability to operate under adverse weather conditions [3]. Furthermore, they are more cost-effective than, for example, LIDAR systems [4]. In the future, the integration of these on-board Advanced Driving Assistance Systems (ADAS) into a wide network of Intelligent Transport Systems (ITS) [5] will increase the security of our roads and streets. This will reduce the probability of traffic accidents and, consequently, minimize the number of victims. Thus, ADAS represents the first link in the electronic security chain configured by ITS, an advanced communication system that provides links and information tools among vehicles, infrastructures and people [6].

Current automotive radar frequency bands range from 76 to 81 GHz [7,8,9], corresponding to wavelengths between 3.94 and 3.70 mm. Formerly, frequencies ranging from 24 to 26 GHz were also considered, which were then repurposed for 5G mobile communications at millimeter-wave frequencies [10]. Moving the radar to higher frequencies gives rise to advantages such as larger bandwidths, better spatial resolution and reduced size.

Radar sensors can be mounted behind the emblem, the front grille, or integrated behind or within the bumper and covered with a radome where necessary [11,12]. Placing sensors behind the bumpers reduces the codependency in the design of both elements, giving the designer more degrees of freedom. A more independent design of both the radar and bumper allows more flexibility in the size of the radar equipment (including the antennas) and improves the overall aesthetic of the bumper [12]. This is desirable due to the increasing number of radars and other sensors that are mounted in various places all around the vehicles. Nevertheless, the impact of transmitting an electromagnetic signal via a bumper in the automotive radar frequency band must be analyzed, as has been carried out in the past for radome emblems [13,14].

With that motivation, we performed a set of measurements to characterize the signal attenuation caused by different samples of car bumpers. This paper is organized into six sections. After this introduction, Section 2 provides an overview of the evolution of automotive radar. In Section 3, we provide a summary of common car bumper materials, paints and finishes, and we introduce the specific characteristics of the bumper samples we have analyzed. Then, in Section 4, the experimental setup and the measurement procedures are described. The measurement results are discussed in Section 5, and finally, we summarize the main conclusions in Section 6.

## 2. Automotive Radar and Autonomous Vehicle

Automotive radar has experienced a major evolution over the last decades as an essential part of the improvement of road safety systems [15], in the trend of increasing the importance of automotive electronics, in general, and ADAS, in particular. From the first road tests in the early seventies, radar-based safety systems have moved from luxury status to manufacturing in series for family vehicles due to the mass production of sensors.

The first frequencies used for automotive radar were around 10 and 16 GHz [16]. However, the antennas had to be large to fulfill the requirement of narrow beam widths. After that, many other different frequencies were tested as follows [17,18]: 35 GHz (selected due to the low atmospheric attenuation), 47 GHz, 60 GHz (especially in Japan) and 94 GHz (as there was military technology previously developed for this band, which reduces the cost of new designs or applications). Finally, the main emphasis was placed on the 24 GHz band, due to the low cost of sensors, and on the 77 GHz band, because of the reduced antenna size and larger bandwidth [16]. This last parameter becomes relevant when the number of sensors increases as more complex modulation schemes can be used to avoid mutual interference and ensure the coexistence of the different systems and vehicles.

The earlier designs were based on Gunn oscillators, Schottky diodes, waveguides and horn antennas. Due to the arrival of GaAs Monolithic Microwave Integrated Circuits (MMICs) that can be integrated with microstrip antennas, more compact and lighter systems were possible [19]. SiGe MMICs allow multiple channels to be integrated into the same circuit, resulting in the incorporation of MIMO capabilities. Specifically, automotive radars have special requirements compared to other mm-wave transceivers, like supporting full-duplex operations in the same frequency band or fast chirping in a broad bandwidth [20].

With the improvements in automotive radar technology, the market of ADAS has continuously grown during the last ten years [16], automating an increasing number of driving tasks. The first application of automotive radar was the detection of vulnerable road users in blind spots. After that, the collision warning systems were followed by distance control and lane change assistance (in combination with image processing systems). In the currently used frequency band, the lowest frequencies (76–77 GHz) are intended for the so-called Long Range Radar (LRR), and the higher frequencies (77–81 GHz) for Short Range Radar (SRR) systems [21]. The LRR band is typically used to develop sensors for adaptive cruise control and collision detection systems, with typical operation ranges of up to 250 m. SRR sensors, on the other hand, are most often used for applications requiring high resolution, such as pedestrian or blind spot detection and lane change assistance.

The requirements established for the automotive radar in the early research projects were 100 m range and 2.5° and 3.5° for azimuth and elevation beam widths, respectively [17]. The currently recommended characteristics for automotive radar in the 77 GHz band include:

- Measurement ranges up to 50 m, 100 m or 250 m (depending on the application, as stated in the introduction);
- Bandwidths up to 1 or 4 GHz (for high-resolution applications);
- Maximum effective isotropic radiated power between 33 and 55 dBm;
- Antenna 3dB-beamwidths (3dB-BW) defined in Table 1 [7,22].

As observed in Table 1, the industry establishes differences between front and corner applications. Front applications comprise functionalities such as adaptive cruise control, and corner applications consist of those like blind spot detection.

For example, current commercial frontal radars manufactured by *Bosch* work in the range from 76 to 77 GHz, with detection ranges up to 302 m (depending on the model) and MIMO capabilities [23]. Bosch corner radars also work in the same frequency range with detection distances up to 160 m [24]. Similar ranges are available in Aptiv’s Gen7 radar family [25]. On its part, Valeo has the MB79, a multi-beam radar sensor working at 79 GHz [26]. In addition, Continental manufactures the SSR520 operating in the frequency range from 76 to 77 GHz, with a detection range of 100 m. In the same frequency band, Continental also offers several LLR radars operating at distances up to 300 m [27]. Probably, the radar from ZF Friedrichshafen is the one on the market with the largest detection range: 350 m [28].

## 3. Car Bumpers Characteristics

Most modern bumpers are produced using polymer materials with different fillers (mainly carbon black and talc). E. B. Mano et al. [29] performed thermal analyses on recycled bumper materials to gain insight into the most commonly used polymer materials. Their research found that most car bumpers are made of a blend of polymers consisting of a large amount of polypropylene (PP) and smaller amounts of high-density polyethylene (HDPE) and ethylene propylene diene methylene (EPDM).

**Figure 1:**
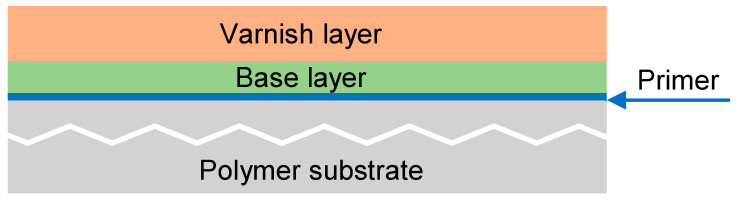
Layer distribution schema of car bumpers.

Besides the material that composes the bumper, the covering paint may also play an important role in radar signal propagation. As discussed by Caddy [30], most modern car paints contain acrylic and/or alkyne resins and cross-linking agents like amino resins, urethanes or styrene. Most car paints are applied in multiple layers. Usually, a layer of primer is followed by a few layers of base coat [31]. This is confirmed in the information provided by the company that supplied the bumper test samples we analyzed. Thus, the car bumper has a layered structure, as shown in Figure 1.

The bumper samples used in this work were kindly provided by an automotive sector company, supplying external components and modules to major car manufacturers. Table 2 provides information on some characteristics of the five samples that were analyzed (labeled as samples A, B, C, D and E). Most of the samples were made of a copolymer of polypropylene (PP) and polyethylene PE, except for sample E, which also contained 10% of talc. Samples C, D and E were painted with a primer layer, a base layer and a layer of colored varnish. Sample A was unpainted, and sample B was painted with a layer of primer, two base layers and a layer of varnish. 

**Figure 2:**
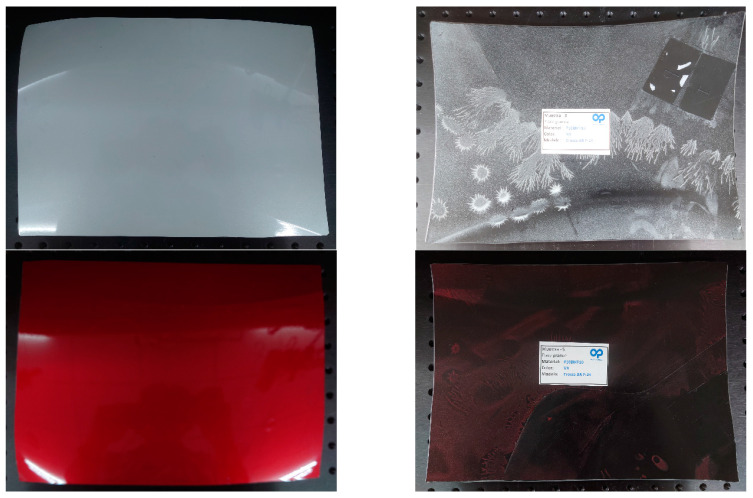
Front- and backsides of bumper samples D (**down**) and E (**up**).

All samples had a thickness of around 4 mm, and the manufacturer provided elements as flat as possible, cut from the most uniform section of the bumper design. They were parts of the current pieces and thus their shapes depend on the car model project. Figure 2 shows some of the samples used.

## 4. Experimental Setup and Procedure

This section begins with a description of the equipment and other resources constituting the measurement setup used in the characterization of the bumper samples. Then, we explain the equipment configuration and the procedure followed in the measurements.

### 4.1. Measurement Setup

**Figure 3:**
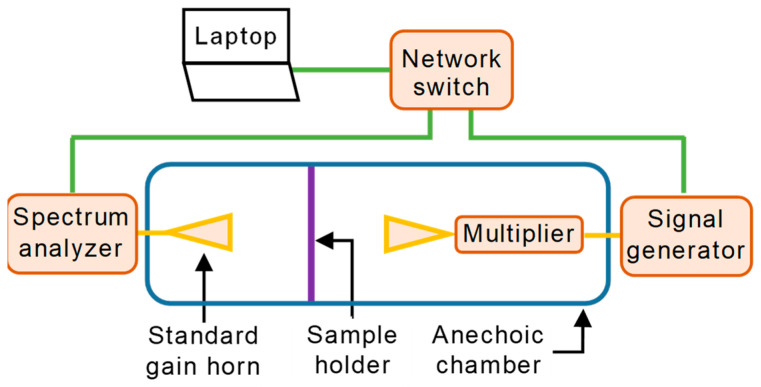
Diagram of the measurement setup.

Figure 3 shows a diagram of the measurement setup. The measurement equipment is highlighted in orange and the antennas are represented by yellow triangles. The green lines correspond to the Ethernet cables, and the yellow lines depict coaxial cables. The anechoic chamber is sketched in blue, and the holder with the material is visible as a purple line between the two antennas. The measurement equipment is connected via a network switch to a laptop to enable remote control.

We used a Rohde & Schwarz SMB100A microwave signal generator to create the transmitted signal at an intermediate frequency. This signal feeds a Rohde & Schwarz SMZ110 frequency multiplier whose output is in the desired radar frequency range. As the frequency multiplier can be connected to the signal generator via the USB, both devices can be configured as one single device. This indicates that we can directly switch the signal generator to the frequency we need at the output of the multiplier.

A Rohde & Schwarz FSW85 signal and spectrum analyzer, operating in the range from 2 Hz to 85 GHz, was the core equipment at the receiver side. Two directional standard gain horn antennas with a gain of 20 dBi completed the setup at both the transmitter and receiver ends.

**Figure 4:**
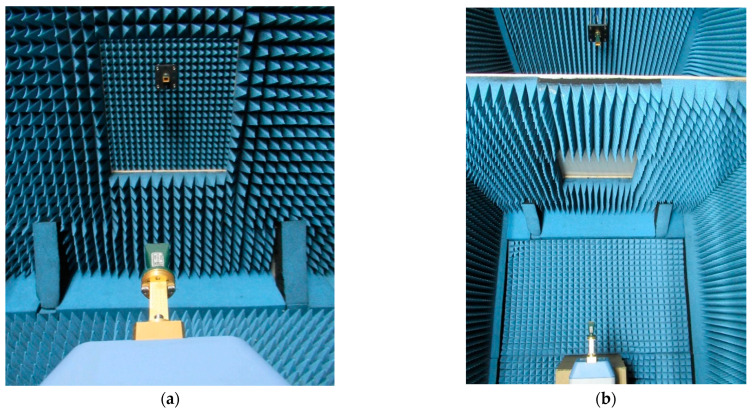
Setup inside the anechoic chamber: (**a**) without sample (view from the transmitter side); (**b**) with sample (top view).

The antennas and the multiplier were placed inside an anechoic chamber [32], as shown in Figure 4, while the rest of the equipment remained outside. The backside of the antennas and the entire multiplier were covered with absorbers to avoid undesired reflections. For clarity, the absorbing material around the antennas and the frequency multiplier were removed when taking the pictures. The absorbers used in the chamber were Eccosorb VHP-2 from Emerson & Cuming, which can be used at 3 GHz. The manufacturer provided data up to 110 GHz, which is the attenuation in the automotive radar frequency band of around −55 dB. The temperature during the measurements is always controlled to be in the range of 21 ± 1 °C to limit its influence on the uncertainty of the measurements [33].

**Figure 5:**
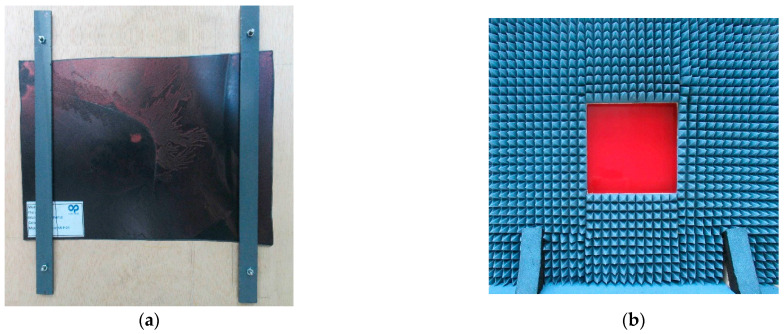
Sample holder, (**a**) receiving side, and (**b**) transmitting side.

The sample holder consisted of a flat panel of wood with a rectangular hole in the center, sizing 20.7 cm by 20.7 cm. The holder with the sample was placed halfway between the two antennas, at a 53 cm distance from each other, meeting the far-field condition of the antennas (15 cm at the highest frequency of the band under study, considering the size of the antennas used. It reached both lateral walls, ceiling and floor of the chamber, thus dividing the anechoic volume into two areas. The hole size was chosen so that the radius of the area illuminated by the 3dB beam width of the antennas had its footprint inside the bumper sample at the selected measurement distance. The center of the hole was aligned with the antenna centers. The bumper samples were attached to the holder on the reception side using four screws and two plastic bands (see Figure 5). For its part, the holder was coated with an absorber on the transmitting side to ensure that the signal received was only passing through the bumper sample. Under the absorber, a metallic film guarantees maximum isolation between both sides of the setup, minimizing the transmission by areas different from those corresponding to the samples under test.

### 4.2. Measurement Procedure

Measurements were performed in the range of 76 GHz to 81 GHz with frequency steps of 1 GHz. First, we set one of the desired frequencies in the signal generator. Then, the same frequency was tuned in the frequency analyzer that was configured in the zero-span mode. Thus, the power level of a single frequency is measured in the time domain, allowing for noise reduction when averaging all measurement points. For this purpose, up to 1001 measurement points were taken at each frequency. The resolution bandwidth was set to 1 kHz. This procedure was repeated for each frequency spot.

The transmitting and receiving frequencies were synchronized by connecting both the signal generator and the spectrum analyzer to the same Rubidium frequency standard. With each frequency change, we provided the system with a warm-up time of several minutes until a stable signal was received. Once the signal was stable, the free-space transmission technique [34,35,36,37] was used to compute the attenuation. First, a reference measurement was taken with the empty sample holder. Then, a bumper sample was mounted in the holder to measure its effect on the propagated signal. Finally, the attenuation induced by the bumper was computed at each frequency as follows:

Attenuation (dB) = Power_free_space_ (dBm) − Power_bumper_ (dBm) (1)

The variable Power_free_space_ represents the power received when there is no sample, and Power_bumper_ averages the power received when the transmitted signal passes through the bumper sample. Both quantities averaged 1001 samples taken at each frequency point.

## 5. Results and Discussion

**Figure 6:**
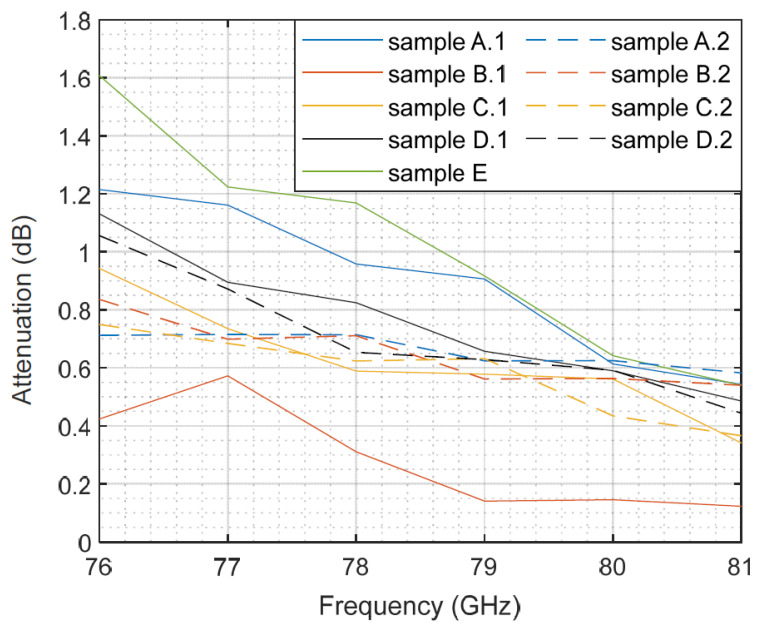
Measured attenuation as a function of the frequency for the different bumper samples.

The first measurements were taken with the samples as provided by the company, i.e., with the actual curvature they had. Although they tried to provide the flatter samples they could, it was almost impossible to find a perfectly flat section in a real-world modern car bumper. For each type of composition (A, B, etc.), two samples were measured (A.1 and A.2, B.1 and B.2, etc.), except for sample E, as only one piece was available. The measured attenuation for each bumper sample is depicted in Figure 6. The horizontal axis represents the frequency, and the vertical axis represents the attenuation in dB. Each colored line depicts the results for a specific sample type. The same color but different line styles (solid or dotted) were used for samples with the same composition. From the figure, we can observe that samples of the same material present more differences in their attenuation compared to others with different compositions. This could be associated with the air gaps between the sample and the holder caused by the curvature that changes among the samples depending on how they are positioned. This seems to cause variability that compromises the accuracy of the measurements.

**Figure 7:**
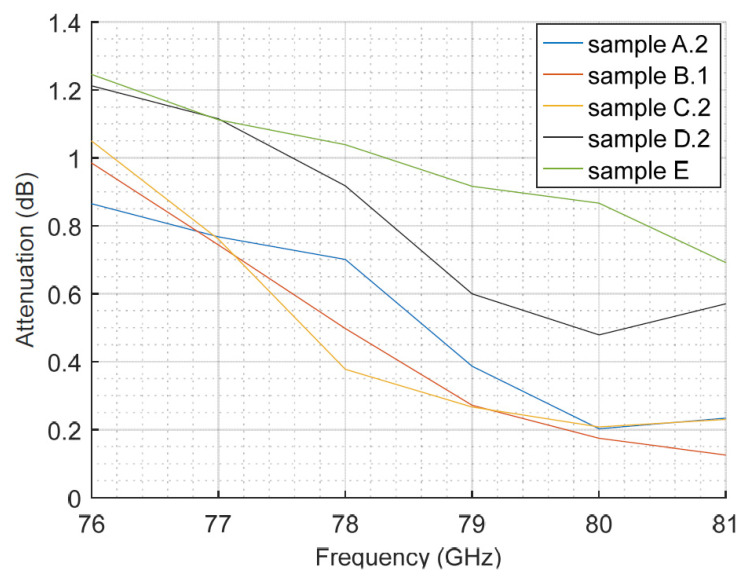
Measured attenuation as a function of the frequency for flat bumper samples.

With the aim of isolating both effects, the curvature and the composition itself, we flattened one sample of each bumper type and then repeated the measurements. The measurement outcomes for the flat samples are shown in Figure 7. The results show a decreasing trend of the attenuation with the frequency, with reductions between 0.55 dB and 0.86 dB when comparing the end frequencies in the radar band. This result agrees with the transmission measurements for painted polycarbonate samples described in [38] for the same frequency band. 

If we compare the results of the curved and flat samples, despite the differences, the trend and order of magnitude are the same. Thus, the rest of the analysis focuses on the flat samples.

The maximum attenuation observed in the flat samples is 1.25 dB. Thus, the propagated radar signal would suffer an additional attenuation lower than 2.50 dB when passing through the bumpers (considering the out and back paths in the monostatic radar configuration typical for these car devices). This is translated into a needed increase in the transmitted power (by means of higher gain amplifiers and/or antennas) to maintain the same detection range, or into a reduction in the detection range when maintaining the same antennas and amplifiers.

The reduction in detection distance can be calculated using the radar range equation, which relates the power delivered to the receiver with the transmitted power within a radar system. Its simplest form for a monostatic radar is [39],

(2) $$P_{r}=\frac{P_{t}G^{2}\lambda^{2}\sigma }{{\left(4\pi \right)}^{3}R^{4}}$$

where

- $P_{r}$ is the received power;
- $P_{t}$ is the transmitted power;
- G is the gain of the antenna (transmitting and receiving antenna is the same);
- λ is the wavelength;
- σ is the radar cross-section of the target;
- R is the distance between the radar and the target.

The power received at a distance reduced in $R^{\prime }$ would be,

(3) $${P^{\prime }}_{r}=\frac{P_{t}G^{2}\lambda^{2}\sigma }{{(4\pi )}^{3}{\left(R-R^{\prime }\right)}^{4}}$$

Then,

(4) $$\frac{{P^{\prime }}_{r}}{P_{r}}=\frac{R^{4}}{{\left(R-R^{\prime }\right)}^{4}}$$

As in our case the power is reduced by 2.5 dB,

(5) $$10\log_{10}\frac{R^{4}}{{\left(R-R^{\prime }\right)}^{4}}=2.5$$

(6) $$R^{\prime }=R\;\left(1-10^{\frac{-2.5}{40}}\right)$$

Then, the detection distance is reduced by 33.5 m for radars configured to work at distances of 250 m. In the same way, those configured to 50 m and 100 m would suffer a decrease of 6.7 m and 13.4 m in their range, respectively. However, as reported in Section 2, commercial radars achieving detection distances of 300 m, or even more, have enough transmission power to compensate for the attenuation caused by the bumpers, keeping the range within requirements.

There are several factors that affect the attenuation of the car bumper, such as the material and the thickness of the coating, and so on. In this work, the analysis of all of them should be limited to the available samples; they come from real-world automotive elements, not from a chemical company that could build samples with varied and specific compositions, thickness and coatings. Taking into account these contour conditions, and as shown in Figure 7, we can observe these factors affecting the attenuation. Thus, bumper samples B.1 and C.2 show quite similar values. However, sample B.1 has two layers of base and sample C.2 only has one, so the base thickness does not seem to clarify the attenuation effect. This idea is reinforced by the performance of sample A.2, which is not painted and shows similar or even higher attenuation values than those of the two samples mentioned earlier. This also occurs in the simulation results in [40], where the losses due to a non-painted bumper can be higher than those of painted pieces. In that work, we can also see values in the order of the ones we have measured. Reference [41] describes that the metallic paint permittivity shows an imaginary part higher than that of the pearlescent paint, and that both have higher values than solid paint. Hence, the paint composition could explain why the attenuation of sample C.2 is almost the same as that of sample B.1, despite having only one layer of base.

The slightly thicker varnish layer of bumper samples D.2 and E could explain the higher attenuation than the others. Sample E has a similar behavior compared to sample D.2 at lower frequencies, but it presents higher attenuation values in the upper part of the analyzed frequency band. Bumper sample number E has a composition of 10% talc. According to [42], the real part of the permittivity increases with the concentration of talc in the substrate. However, the paint composition of sample E seems to compensate for this effect and still presents the highest attenuation among the samples. 

**Figure 8:**
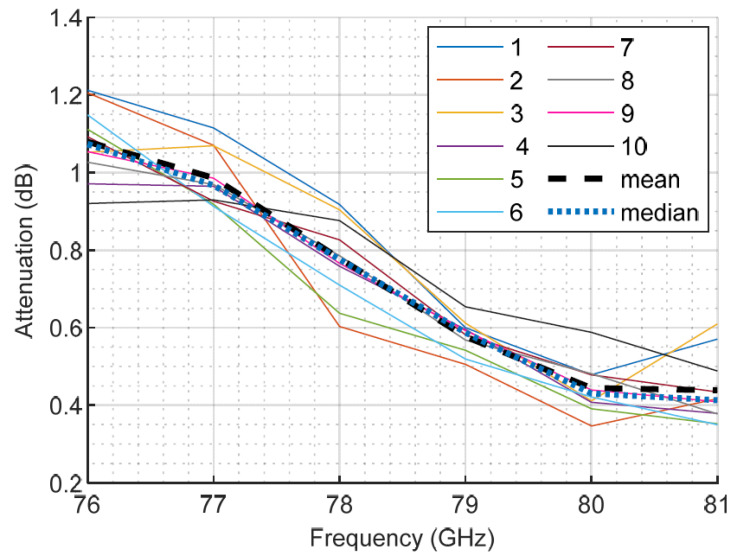
Results of several measurements of sample D.2.

To validate the accuracy of the results obtained, one of the bumper samples (labeled as D.2) was measured 10 times. Each of the measurement results is plotted in Figure 8, together with their mean and median. The differences between the mean and the median values were negligible, indicating that there were no anomalous results [43]. The confidence of the experimental data was also supported by the statistical parameters of the measurement errors at each frequency, which are summarized in Table 3.

The uncertainty of the measurements is defined in terms of the experimental standard deviation, s, by (7).

(7) $$s=\sqrt{\frac{1}{N-1}{\left(M_{i}-M_{\mathrm{mean}}\right)}^{2}}$$

where N is the number of measurements performed, M_i_ is each individual measurement result and M_mean_ is the mean of the 10 results.

**Figure 9:**
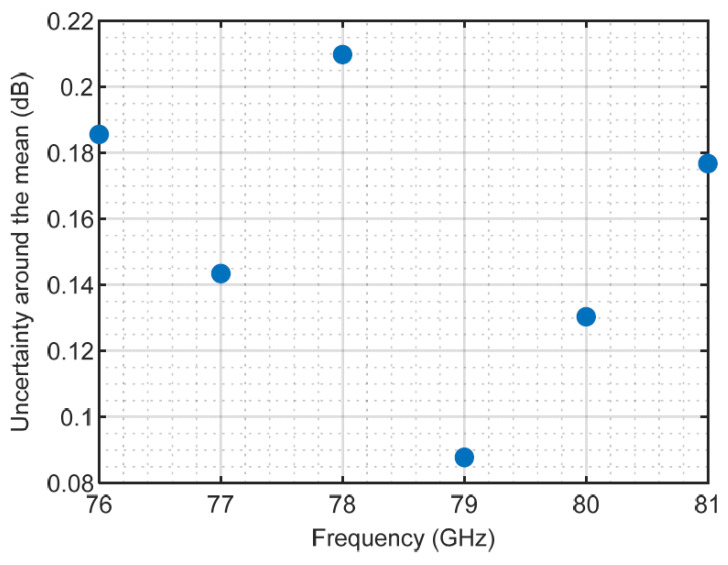
Uncertainty in dB derived from measurements of sample D.2, with a 95% coverage interval.

If we apply a coverage factor of 1.96 (95% of the values falling in the interval [−1.96 s, 1.96 s]), we obtain the values shown in Figure 9.

**Figure 10:**
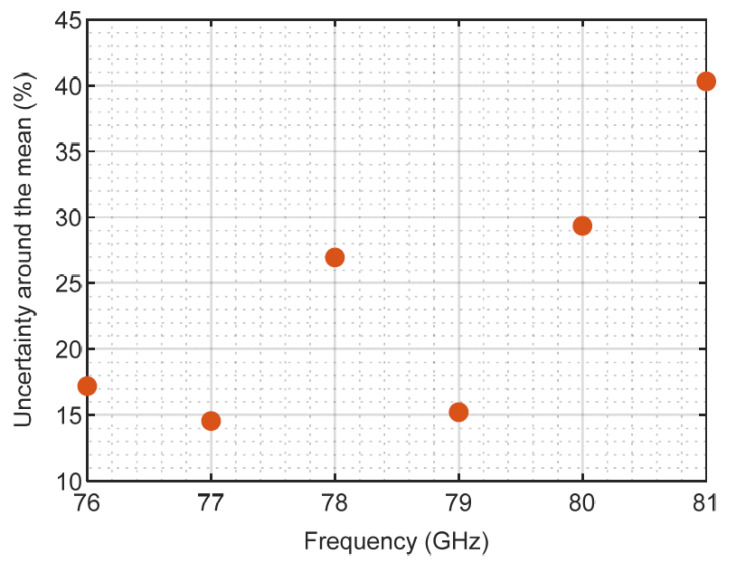
Uncertainty in % derived from measurements of sample D.2, with a 95% coverage interval.

If we calculate the relative uncertainty, i.e., normalized by the mean value (Figure 10), we can observe that, in general, it is more difficult to measure the attenuation with precision as the frequency grows.

These values of uncertainty agree with those reported in [44] for shielding effectiveness measurements in textiles. The free-space transmission method was also used by these researchers, but the equipment used was a vector network analyzer (VNA). In [45,46], uncertainties for attenuation measurements with a spectrum analyzer in building materials are estimated as 0.35 dB and 0.43 dB, respectively. Our uncertainty values are also acceptable compared with other radio-electric measurements in free space. In [47], the uncertainty budget reveals errors of around 40% when the field strength was measured with a spectrum analyzer. Ref. [48] reports the uncertainty values of an antenna gain when measured by different labs. Such uncertainty results are around 0.14 dB in best case scenarios. In summary, the results are in good agreement with those previously published in the literature for comparable experiments.

In addition to the measurements, there has been an attempt to model the attenuation provided by the car bumpers using a multilayer transmission model, including various layers related to the composition of the plastic support and the different superposed layers of painting and varnishing with a variety of chemical compositions [41]. Although the theoretical effort is indeed valuable, the accuracy of the results does not seem to be too confident, and the measured attenuation figures recommend the consideration of something like a guard value in the radio link budget when the transmitter is covered by the plastic bumper.

After discussing the main results obtained, the contents of this manuscript are compared with those of other published works, which are summarized in Table 4.

As the study in [38] is more focused on a methodology to determine the permittivity than on the analysis of real samples, the measurement systems are focused on measuring small parts of the sample for homogeneity. For this reason, the substrate used in this study was not the same material as that used for the bumpers. Polycarbonate (PC) was chosen as the substrate material instead of polypropylene (PP) for homogeneous reasons. Still on the samples issue, the main difference of our study is that measurements were done in curved and flattened bumper samples.

**Figure 11:**
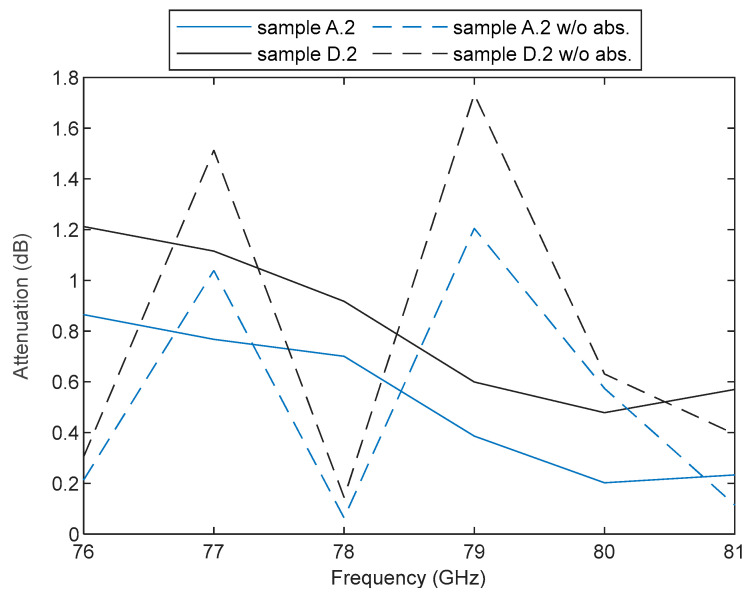
Measured attenuation of bumper samples A.2 and D.2. Dotted lines represent attenuation measurements without absorbers covering the multiplier and the back of the antennas, and the solid lines represent the measurements after adding the absorbers.

In [41], the measurements were performed in a lab environment with absorbers placed only on the wall closest to the antennas to avoid reflections from metallic components. The setup in [42] is also in a lab environment with no absorbers. We decided to use the controlled environment of an anechoic chamber and to cover any element inside it with absorbers. When testing the setup, we experimentally verified that, at the analyzed frequency range, any surface becomes an important source of reflection, even the frequency multipliers. This caused interferences in the received signal, thereby compromising the accuracy of the results. This is better explained in Figure 11, where the effects of measuring two of our samples with and without absorbers around the pieces are presented. This figure helps to support the importance of mitigating any possible source of diffraction within the measurement setup. In addition, the use of absorbers also ensured that there was no transmission around the bumper sample itself. 

The uncertainty study in [41] is limited to error bars in the graphs representing the standard deviations for three individual measurements. However, no numerical results are provided for a clear comparison with the measured values, and the confidence intervals are not defined. The last difference between both studies is the polarization used in the antennas.

## 6. Conclusions

We measured the attenuation that different samples of car bumpers cause in the propagation of the electromagnetic signals at the working frequency band of automotive radar. These results are of interest in a time of strong development of ADAS, in which locally gathered information could be shared in the future, integrated into the wide coverage and rapidly developed ITS technology [5].

Curved and flat bumper samples were analyzed. In the curved samples, more variability was observed, probably due to the curvature itself and the air gaps between the sample and its holder. Despite this, the attenuation trend and their orders of magnitude were the same in both cases. We observed that the attenuation has a decreasing trend with the frequency in all samples evaluated, with values below 1.25 dB in the whole band (or 1.44 dB if we consider the uncertainty) for the flat samples. Such values of attenuation imply a reduction in the detection range of 6.7 m, 13.4 m and 33.5 m for radars configured to range for detection distances of 50 m, 100 m and 250 m, respectively, if the penetration losses could not be compensated by increasing the transmitted signal.

Samples with a thicker varnish layer led to higher attenuation values. Also, the paint composition could explain the attenuation differences among the samples, due to the higher imaginary part of the permittivity of paints that contain metallic particles. 

Measurement uncertainties are also good indicators of experimental confidence. The uncertainty analysis showed an acceptable accuracy of the results of the flat samples. It was limited to 0.21 dB with a 95% coverage interval in the measured frequency band. The lower the frequency in the measured band, the more accurate the measurements seemed to be (with uncertainties around 15% in the observed band). The values obtained for uncertainty are in good agreement with other measurements of materials using the free space technique and also with other radio-electric measurements in free space, as found in the literature.

In recent years, the use of automotive radar bands for communication with other vehicles or infrastructure has been proposed [6]. The attenuation values provided in this work would also be useful for implementing such joint communication radar systems, in case the evolution of ITS leads to these frequencies.

## Figures and Tables

**Table 1:** 3dB-beamwidths for automotive radar antennas.

Type of Radar	Azimuth 3dB-BW		Elevation 3dB-BW	
	Tx	Rx	Tx	Rx
Radar A. Automotive radar. Front applications (250 m).	5°	5°	3°	3°
Radar B. Automotive high-resolution radar. Front applications (100 m).	12.5°	13.5°	5.5°	5.5°
Radar C. Automotive high-resolution radar. Corner applications (100 m).	12.5°	16°	5.5°	5.5°
Radar D. Automotive high-resolution radar. (100 m).	16°	16°	5.5°	5.5°
Radar E. Automotive high-resolution radar. Very short range applications (50 m).	27°	27°	5.5°	5.5°

**Table 2:** Composition of the car bumper samples analyzed.

Sample	Plastic Type	Type of Paint	Color	Primer Thickness (μm)	Base Thickness (μm)	Varnish Thickness (μm)	Sample Size W (cm) × L (cm)
A	PP and PE copolymer	No paint	FXT	--	--	--	21.9 × 29.8
B	PP and PE copolymer	Three layer	N9 blanc nacre	2–3	27–29	35–37	21.9 × 30.4
C	PP and PE copolymer	Double layer	9V noir pearl black	2–3	12–14	31–33	23.3 × 30.5
D	PP and PE copolymer	Double layer	VH rouge elixir	2–3	19–21	39–41	21.6 × 29.9
E	PP and PE copolymer + talc	Double layer	F4 artense grey	2–3	12–14	41–43	21 × 31

**Table 3:** Statistics of the measurement error.

Frequency (GHz)	Mean Error (dB)	Median of the Error (dB)	Variance of the Error (dB)
76	0.07	0.06	0.003
77	0.06	0.06	0.001
78	0.08	0.08	0.004
79	0.03	0.03	0.001
80	0.05	0.04	0.002
81	0.07	0.06	0.003

**Table 4:** Comparison with previously published works.

Reference	Automotive Radar Frequency Range	Sample Type	Antennas Polarization	Measurement Scenario	Measurement Uncertainty
[38]	76–81 GHz	Painted polycarbonate samples	Not specified	Material Characterization Kit Optical free-space measurement	Not specified
[41] 2020	76–81 GHz	Samples with three kinds of paints (shape and origin not specified)	Horizontal	Free space method Lab environment Absorber in one wall	Error bars in graphs representing the standard deviations
[42] 2017	76–81 GHz	Actual bumper samples	Not specified	Free space method Lab environment No absorbers	Not specified
Our study	76–81 GHz	Curved and flat actual bumper samples	Vertical	Free-space method Anechoic chamber Absorbers around the sample	Yes

## Data Availability

Not applicable.

## References

[1] Marzbani H., Khayyam H., To C.N., Quoc Đ.V., Jazar R.N. (2019). Autonomous Vehicles: Autodriver Algorithm and Vehicle Dynamics. IEEE Trans. Veh. Technol..

[2] Dai Y., Lee S. (2020). Perception, Planning and Control for Self-Driving System Based on On-board Sensors. Adv. Mech. Eng..

[3] Magosi Z.F., Li H., Rosenberger P., Wan L., Eichberger A. (2022). A Survey on Modelling of Automotive Radar Sensors for Virtual Test and Validation of Automated Driving. Sensors.

[4] Sohail M., Khan A.U., Sandhu M., Shoukat I.A., Jafri M., Shin H. (2023). Radar sensor based machine learning approach for precise vehicle position estimation. Sci. Rep..

[5] Maaloul S., Aniss H., Mendiboure L., Berbineau M. (2022). Performance Analysis of Existing ITS Technologies: Evaluation and Coexistence. Sensors.

[6] Ma D., Shlezinger N., Huang T., Liu Y., Eldar Y.C. (2020). Joint Radar-Communication Strategies for Autonomous Vehicles: Combining Two Key Automotive Technologies.

[7] (2018). Systems Characteristics of Automotive Radars Operating in the Frequency Band 76–81 GHz for Intelligent Transport Systems Applications.

[8] (2016). Electromagnetic Compatibility and Radio Spectrum Matters (ERM); Short Range Devices; Transport and Traffic Telematics (TTT); Short Range Radar Equipment Operating in the 77 GHz to 81 GHz Band; Harmonised Standard Covering the Essential Requirements of Article 3.2 of Directive 2014/53/EU.

[9] (2017). Intelligent Transport Systems (ITS); Radiocommunications Equipment Operating in the 5855 MHz to 5925 MHz Frequency Band; Harmonised Standard Covering the Essential Requirements of Article 3.2 of Directive 2014/53/EU.

[10] (2022). 5G.; NR.; User Equipment (UE) Radio Transmission and Reception; Part 2: Range 2 Standalone.

[11] (2019). Anritsu Corporation; Fraunhofer FHR. E-Band Based Car Radar Emblem Measurements. Application Note 11410-01152, Rev. A. https://dl.cdn-anritsu.com/en-us/test-measurement/files/Application-Notes/Application-Note/11410-01152A.pdf.

[12] Miscia M. (2022). Analysis on the Installation of Long-Range Radar Sensors in Modern Vehicles. Master’s Thesis.

[13] Heuel S., Koeppel T., Ahmed S. (2018). Evaluating 77 to 79 GHz Automotive Radar Radome Emblems. Microw. J..

[14] Matsuzawa S.I., Watanabe T. Influence of resin cover on antenna gain for automotive millimeter wave radar. Proceedings of the International Symposium on Antennas and Propagation (ISAP).

[15] Abd El-Hameed A.S., Ouf E.G., Elboushi A., Seliem A.G., Izumi Y. (2023). An Improved Performance Radar Sensor for K-Band Automotive Radars. Sensors.

[16] Waldschmidt C., Hasch J., Menzel W. (2021). Automotive Radar—From First Efforts to Future Systems. IEEE J. Microw..

[17] Meinel H.H. Evolving automotive radar—From the very beginnings into the future. Proceedings of the 8th European Conference on Antennas and Propagation (EuCAP).

[18] Norouzian F., Hoare E.G., Marchetti E., Cherniakov M., Gashinova M. Next Generation, Low-THz Automotive Radar—The Potential for Frequencies above 100 GHz. Proceedings of the 20th International Radar Symposium (IRS).

[19] Walden M. Automotive Radar—From Early Developments to Self-Driving Cars. Proceedings of the ARMMS RF and Microwave Society Conference.

[20] Hung C.-M., Lin A.T., Peng B., Wang H., Hsu J.-L., Lu Y.-J., Hsu W., Zhan J.-H.C., Juan B., Lok C.-H. 9.1 Toward Automotive Surround-View Radars. Proceedings of the International Solid-State Circuits Conference (ISSCC).

[21] Takeda Y., Fujibayashi T., Yeh Y.-S., Wang W., Floyd B. A 76- to 81-GHz transceiver chipset for long-range and short-range automotive radar. Proceedings of the IEEE MTT-S International Microwave Symposium (IMS).

[22] (2012). Millimetre Wave Vehicular Collision Avoidance Radars and Radiocommunication Systems for Intelligent Transport System Applications.

[23] Robert Bosch GmbH (2023). Front Radar Sensor. https://www.bosch-mobility-solutions.com/en/solutions/sensors/front-radar-sensor/.

[24] Robert Bosch GmbH (2023). Corner Radar Sensor. https://www.bosch-mobility-solutions.com/en/solutions/sensors/corner-radar-sensor/.

[25] Aptiv PLC (2023). Gen 7 Radar Family. https://www.aptiv.com/en/gen7-radar-family.

[26] Valeo S.A. (2023). Valeo.ai. https://www.valeo.com/en/valeo-ai/.

[27] Continental AG (2023). Radars. https://www.continental-automotive.com/en-gl/Passenger-Cars/Autonomous-Mobility/Enablers/Radars.

[28] ZF Friedrichshafen AG (2023). Imaging Radar. https://www.zf.com/products/en/cars/products_64255.html.

[29] Mano E.B., Martins A.F., Mendes L.C. (2000). Thermal Analysis Applied to Discarded Car Bumpers. J. Thermal Anal. Calorim..

[30] Caddy B. (2001). Forensic Examination of Glass and Paint.

[31] Zieba-Palus J. (2020). Examination of the variation of chemical composition and structure of paint within a car body by FT-IR and Raman spectroscopies. J. Mol. Struct..

[32] Expósito I., García Sánchez M., Cuiñas I. (2020). Uncertainty Assessment of a Small Rectangular Anechoic Chamber: From Design to Operation. IEEE Trans. Antennas Propag..

[33] Expósito I., García Sánchez M., Cuiñas I. (2019). Computing the Influence of Environmental Conditions in Electromagnetic Measurements Uncertainty. IEEE Trans. Antennas Propag..

[34] García Sánchez M., Iglesias C., Cuiñas I., Expósito I. (2022). Building Penetration Losses at 3.5 GHz: Dependence on Polarization and Incidence Angle. Electronics.

[35] Zhekov S.S., Nazneen Z., Franek O., Pedersen G.F. (2018). Measurement of Attenuation by Building Structures in Cellular Network Bands. IEEE Antennas Wireless Propag. Lett..

[36] Ferreira D., Caldeirinha R.F.S., Fernandes T.R., Cuiñas I. (2018). Hollow Clay Brick Wall Propagation Analysis and Modified Brick Design for Enhanced Wi-Fi Coverage. IEEE Trans. Antennas Propag..

[37] Kowal M., Kubal S., Zielinski R.J. Measuring the shielding effectiveness of large textile materials in an anechoic chamber. Proceedings of the International Symposium on Electromagnetic Compatibility—EMC Europe.

[38] Winter C., Korff M., Fabbri T., Holzknecht S., Weber I., Biebl E.M. (2022). Permittivity Determination Method for Multilayer Automotive Coatings for Radar Applications at 77 GHz. IEEE Trans. Microw. Theory Technol..

[39] Balanis C.A. (2008). Modern Antenna Handbook.

[40] Dash J.C., Sarkar D., Antar Y. Effect of Painted Bumper on Automotive MIMO RADAR Performance Study Using Bi-directional Loss and Antenna Array Ambiguity Function. Proceedings of the IEEE International Symposium on Antennas and Propagation and USNC-URSI Radio Science Meeting (AP-S/URSI).

[41] Xiao Y., Norouzian F., Hoare E.G., Marchetti E., Gashinova M., Cherniakov M. (2020). Modeling and Experiment Verification of Transmissivity of Low-THz Radar Signal Through Vehicle Infrastructure. IEEE Sensors J..

[42] Emilsson E.P. (2017). Radar Transparency and Paint Compatibility: A Study of Automobile Bumper and Bumper-Skin Complex Permittivities for 77GHz Microwaves. Master’s Thesis.

[43] (2022). Statistical Methods for Use in Proficiency Testing by Interlaboratory Comparison.

[44] Patel S.M., Patel K., Negi P.S., Ojha V.N. (2013). Shielding effectiveness measurements and uncertainty estimation for textiles by a VNA-based free space transmission method. Int. J. Metrol. Qual. Eng..

[45] Pavlík M., Gladyr A., Zbojovský J. Comparison of Measured and Simulated Data of Shielding Effectiveness, Reflection and Absorption of Electromagnetic Field. Proceedings of the IEEE Problems of Automated Electrodrive, Theory and Practice (PAEP).

[46] Zbojovský J., Pavlík M., Čonka Z., Kruželák L., Kosterec M. Influence of shielding paint on the combination of building materials for evaluation of shielding effectiveness. Proceedings of the 19th International Scientific Conference on Electric Power Engineering (EPE).

[47] (2019). Basic Standard on Measurement and Calculation Procedures for Human Exposure to Electric, Magnetic and Electromagnetic Fields (0 Hz–300 GHz).

[48] Saporetti M.A., Foged L.J., Svensson B., Alexandridis A., Expósito Pérez I., Álvarez López Y., Tercero F., Culotta López C., Sierra Castañer M. Recent Developments in International Facility Comparison Campaigns. Proceedings of the 41st Annual Meeting and Symposium of the Antenna Measurement Techniques Association.

